# Saccular abdominal aortic aneurysm in adolescence with tuberous sclerosis

**DOI:** 10.1002/ccr3.8715

**Published:** 2024-03-31

**Authors:** Takumi Umibe, Hironobu Nishiori, Shintaro Koizumi, Hiroki Ikeuchi, Goro Matsumiya

**Affiliations:** ^1^ School of Medicine Chiba University Chiba Japan; ^2^ Department of Cardiovascular Surgery Chiba University Hospital Chiba Japan

**Keywords:** abdominal aortic aneurysm, aortic repair, aortic replacement, tuberous sclerosis

## Abstract

**Key clinical message:**

Abdominal aortic aneurysm complicated by tuberous sclerosis is rare, particularly in patients over the age of 10. It is important to screen for abdominal aortic aneurysm in adolescents diagnosed with tuberous sclerosis regularly.

**Abstract:**

A 15‐year‐old girl who was diagnosed with tuberculous sclerosis complicated with a saccular aortic abdominal aneurysm (AAA), measuring 19 × 18 mm in diameter. The patient underwent open repair of AAA using a 11 mm straight prosthetic graft. It is important to screen for AAA in adolescents diagnosed with tuberous sclerosis regularly.

## 1 CASE

**FIGURE 1:**
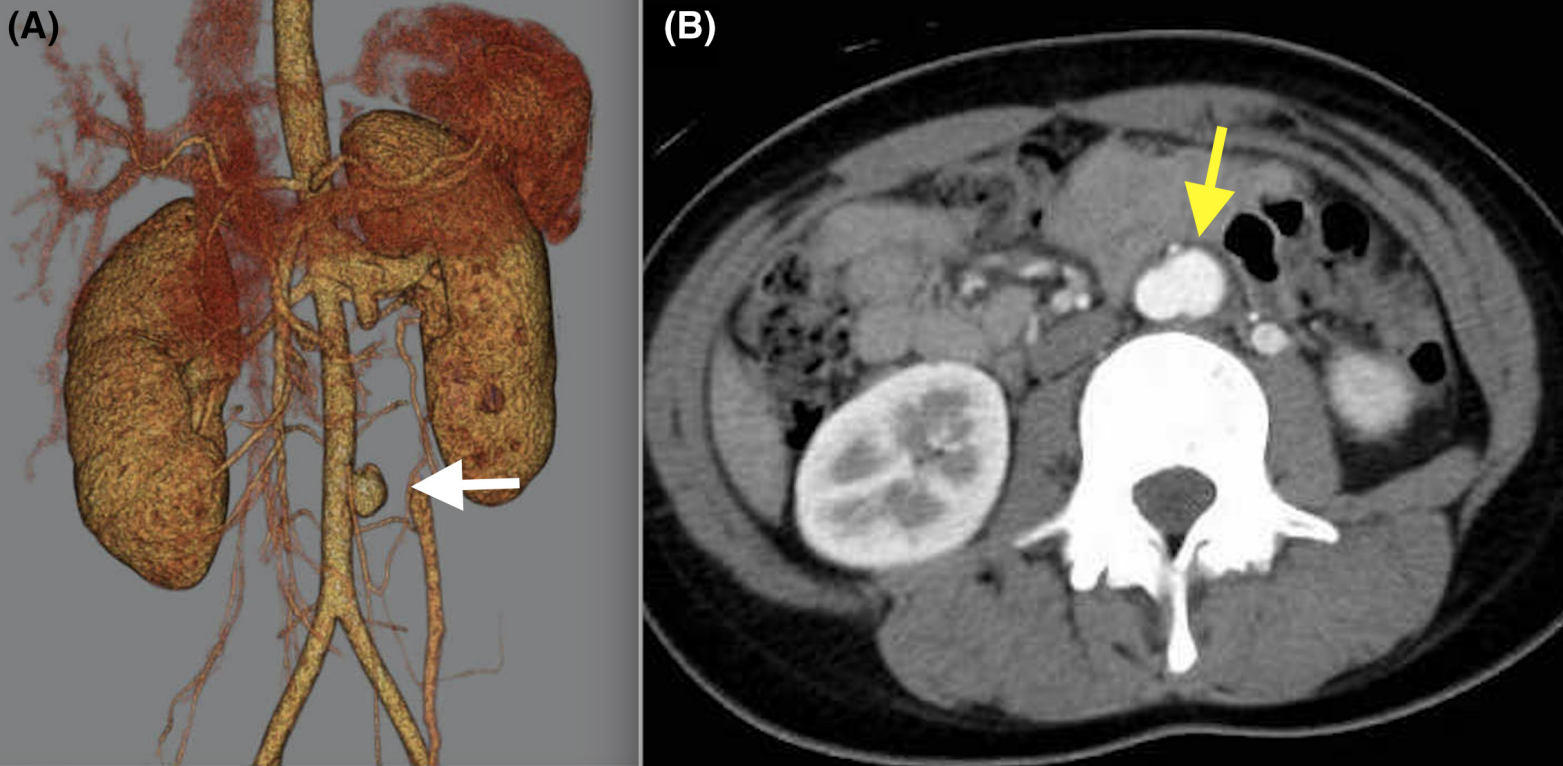
The 3D computed tomography showed an infrarenal abdominal aortic aneurysm of 19 × 18 mm (white arrow). (A), Computed tomography showed a saccular abdominal aortic aneurysm on the left side of the infrarenal abdominal aorta (yellow arrow) (B).

**FIGURE 2:**
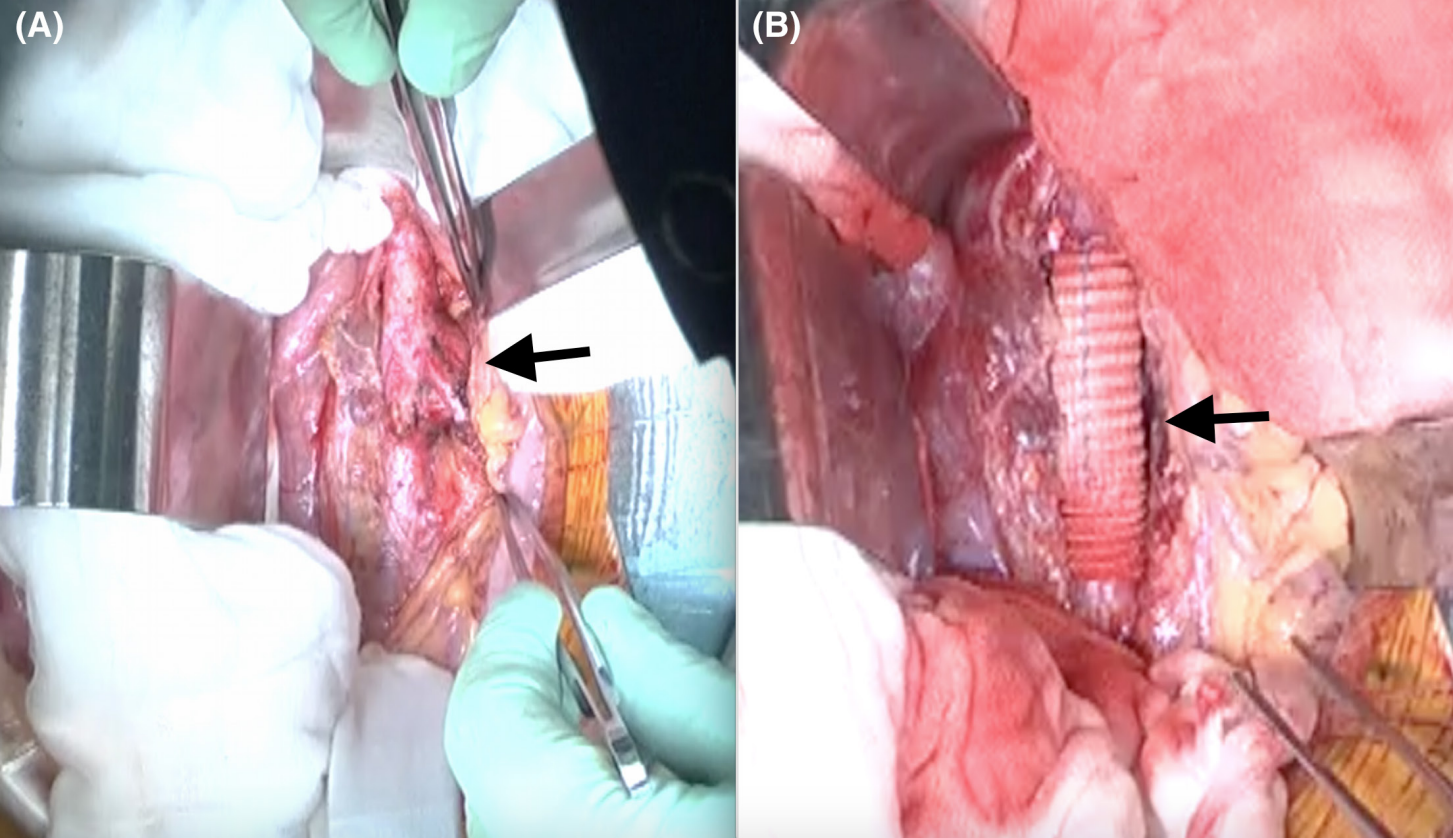
The intraoperative finding of the abdominal aortic aneurysm (black arrow). (A), The abdominal aortic aneurysm was repaired using an 11 mm graft (black arrow). (B).

**FIGURE 3:**
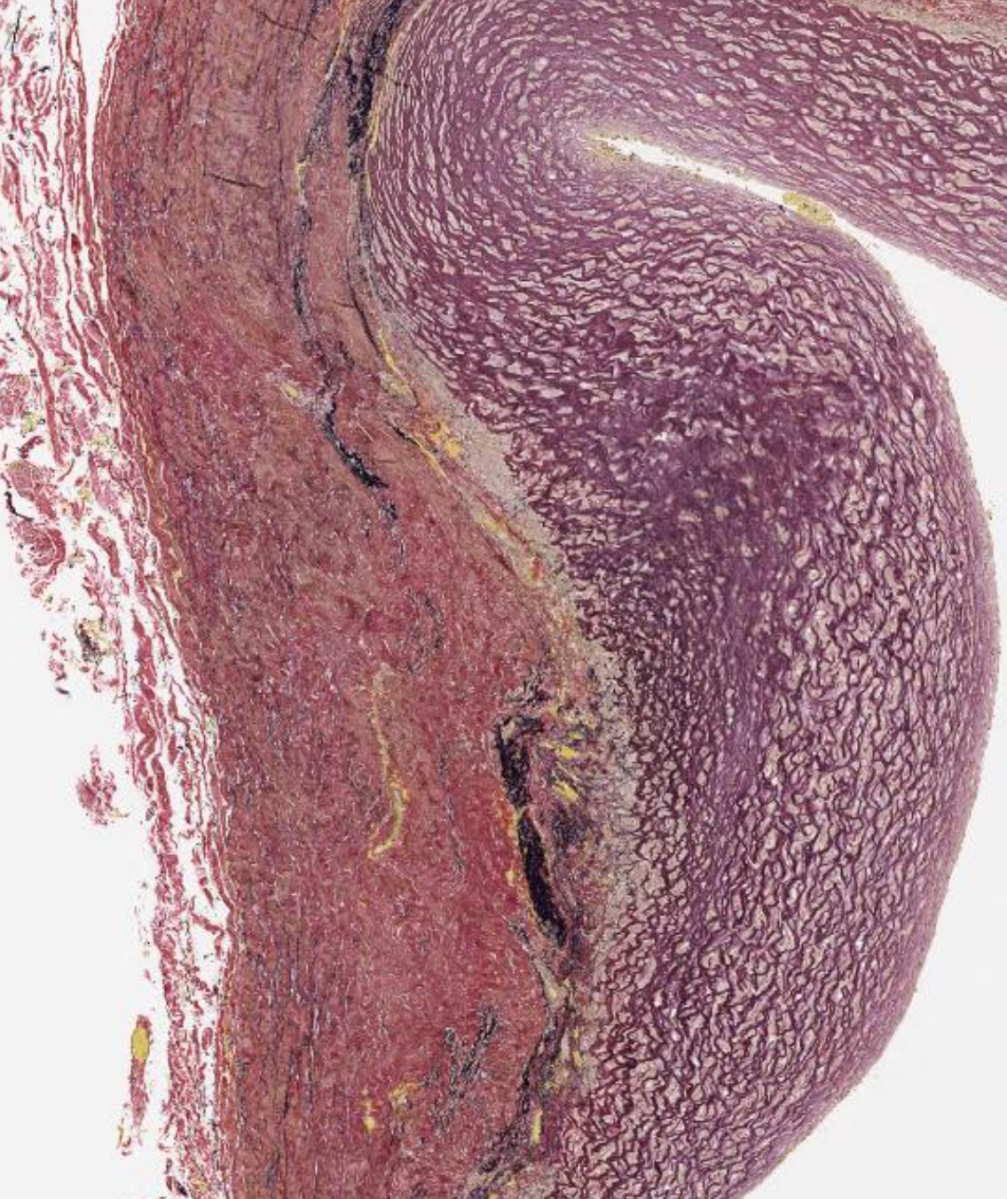
Histopathological picture of the wall of the aneurysm showed thicken intima with fibrosis, medial atrophy, and rough smooth muscle cells with increased fibrous stroma.

A 15‐year‐old female with a history of tuberculous sclerosis (TS) referred to our hospital for surgical management of an abdominal aortic aneurysm (AAA). The computed tomography showed an infrarenal saccular AAA, measuring 19 × 18 mm in diameter.(Figure 1A,B). The patient underwent open repair of AAA using a straight prosthetic graft (Figure 2A,B). The biopsy of the wall of the aortic aneurysm showed thicken intima with fibrosis, ruptured media with increased fibrous stroma(Figure 3). Follow‐up CT 1 year postoperatively revealed a patent graft with no evidence of new aneurysm formation.

Abdominal aortic aneurysm complicated by TS is very rare, particularly in patients over the age of 10.^1^, ^2^ As AAA in TS patients is frequently overlooked, and often diagnosed only after rupture, screening with abdominal echocardiography or computed tomography is crucial. Given that AAA can develop during adolescence, it is important to screen for AAA in adolescents diagnosed with TS regularly.

## AUTHOR CONTRIBUTIONS

**Takumi Umibe:** Data curation; writing – original draft; writing – review and editing. **Hironobu Nishiori:** Supervision. **Shintaro Koizumi:** Supervision. **Hiroki Ikeuchi:** Supervision. **Goro Matsumiya:** Supervision.

## FUNDING INFORMATION

None.

## CONFLICT OF INTEREST STATEMENT

None.

## ETHICS STATEMENT

None.

## CONSENT

Written informed consent was obtained from the patient to publish this report in accordance with the journal's patient consent policy.

## Data Availability

None.
